# A novel approach for denoising electrocardiogram signals to detect cardiovascular diseases using an efficient hybrid scheme

**DOI:** 10.3389/fcvm.2024.1277123

**Published:** 2024-04-04

**Authors:** Pingping Bing, Wei Liu, Zhixing Zhai, Jianghao Li, Zhiqun Guo, Yanrui Xiang, Binsheng He, Lemei Zhu

**Affiliations:** ^1^ Hunan Provincial Key Laboratory of the Research and Development of Novel Pharmaceutical Preparations, Changsha Medical University, Changsha, China; ^2^ College of Mechanical and Electrical Engineering, Beijing University of Chemical Technology, Beijing, China

**Keywords:** electrocardiogram signal, noise removal, S-transform, bi-dimensional empirical mode decomposition, non-local means

## Abstract

**Background:**

Electrocardiogram (ECG) signals are inevitably contaminated with various kinds of noises during acquisition and transmission. The presence of noises may produce the inappropriate information on cardiac health, thereby preventing specialists from making correct analysis.

**Methods:**

In this paper, an efficient strategy is proposed to denoise ECG signals, which employs a time-frequency framework based on S-transform (ST) and combines bi-dimensional empirical mode decomposition (BEMD) and non-local means (NLM). In the method, the ST maps an ECG signal into a subspace in the time frequency domain, then the BEMD decomposes the ST-based time-frequency representation (TFR) into a series of sub-TFRs at different scales, finally the NLM removes noise and restores ECG signal characteristics based on structural self-similarity.

**Results:**

The proposed method is validated using numerous ECG signals from the MIT-BIH arrhythmia database, and several different types of noises with varying signal-to-noise (SNR) are taken into account. The experimental results show that the proposed technique is superior to the existing wavelet based approach and NLM filtering, with the higher SNR and structure similarity index measure (SSIM), the lower root mean squared error (RMSE) and percent root mean square difference (PRD).

**Conclusions:**

The proposed method not only significantly suppresses the noise presented in ECG signals, but also preserves the characteristics of ECG signals better, thus, it is more suitable for ECG signals processing.

## 1 Introduction

The Electrocardiogram (ECG) is a powerful tool to reflect the state of cardiovascular health, and is currently extensively applied in the diagnosis of cardiac diseases. In reality, the ECG is usually susceptible to various types of noises and artifacts (1–5), including power line interference (PLI), Gaussian noise, baseline wander (BW), electrode motion noise (EM) and muscle artifacts (MA), which severely distort the ECG signal and bring more challenging for proper treatment of patients.

To extract the correct information associated with physiology of the heart, various techniques have been proposed toward removing the content of noise in the ECG signals, such as Wiener filter, adaptive filtering, wavelet transform (WT), independent component analysis (ICA), principal component analysis (PCA), empirical mode decomposition (EMD), variational mode decomposition (VMD) and non-local mean (NLM). The key idea of Wiener filter is to minimize the energy spectral density between the target signal and the measured signal (6), which was previously used in stationary signal analysis (7, 8). Since Wiener filter does not need extra sensor information with noisy ECG signals, it has been applied in removal of noise from the ECG signals (9). However, the denoising performance of Wiener filter is not ideal for ECG signals, as the ECG signal is non-stationary. Adaptive filtering makes the denoised ECG signal close to the reference signal by minimizing the mean square error (10, 11). It has been used to suppress motion artifact, electromyogram, power line interference, and baseline wander. It is worth noting that adaptive filtering might be less efficient due to the effect of error in the reference signal that is required, thus, it is difficult to deal with some continuous vibration artifacts (12). Wavelet transform is a popular ECG denoising technique. It can remove the noise from the ECG signals by using the characteristics of noise in the frequency domain (13, 14). Although wavelet transform has many advantages over traditional filtering algorithms, there are still some drawbacks. First, it fails to preserve the edges of the ECG signals. Second, a trade-off exists between accuracy and computational efficiency. Third, the choice of the basis function is also a troublesome task (15). Independent component analysis is a method suitable for separating independent components from ECG complex signals, and principal component analysis is able to reduce dimensionality for feature extraction of the ECG data (16). ICA and PCA denoise the in-band artifacts and noise of the ECG signals by removing the dimensions that correspond to noise (17). Both of them do not produce good results with single-lead ECG recording because they are based on correlation and uncorrelation ideas (18). Empirical mode decomposition is an adaptive and efficient decomposition method capable of decomposing an ECG signal into a series of finite intrinsic mode function (IMF) (19), which is suitable for analyzing nonlinear and non-stationary signals. Denoising by the EMD is usually is achieved by removing lower-order IMFs based on the assumption that the signal and noise are well-separated in frequency bands (20). Nevertheless, for ECG signals, although most signals are concentrated in lower frequencies, the QRS complex spreads across the mid-high frequency bands. Therefore, EMD only reduces the noise but cannot completely remove the noise from the ECG signals. In addition, EMD suffers from an important defect, named mode mixing. To address the problem, several variants of EMD were proposed, e.g., ensemble empirical mode decomposition (EEMD) (21) and complete ensemble empirical mode decomposition (CEEMD) (22). Variational mode decomposition is an enhanced version of EMD (23), which can decompose a noisy ECG signal into a set of narrow-band variational mode functions (VMFs), and then the noise from these narrow-band VMFs is filtered out. The main advantage of VMD is to solve the mode mixing effectively. Furthermore, VMD also provides some useful features such as phase angle that helps to classify the heart rhythm with abnormalities (24, 25). Non-local means is an effective image processing technique by averaging the different regions with similar features (26). The NLM is capable of preserving edge information, but it relies too much on the local width and half width of neighborhood. Therefore, the denoising performance of NLM is reduced with the increasing noise in the ECG signals.

The aforementioned techniques are mainly based on the difference between ECG signal and noise in the time domain, frequency domain or time-frequency domain for denoising ECG signals. However, such methods often have limitations in characterizing the deeper feature differences between ECG signal and noise. The main aim of this study is to propose a novel denoising method, named multi-scale time-frequency decomposition, to remove noise in ECG signals, where the characteristics of ECG signal and noise are refined and easier to identify in the multi-scale time-frequency domain. It combines S-transform (ST), bidimensional empirical mode decomposition (BEMD) and non-local means (NLM). Within this method, BEMD decomposes time-frequency map from ST into sub-time-frequency map of different scales, and then NLM is employed to eliminate noise at different scales. The proposed method is evaluated in various noises such as PLI, Gaussian noise, BW, EM and MA. The major contributions of this paper are summarized as follows:

(1) We present a robust and efficient time-frequency denoising framework for noise removal in ECG signals.
(2) The presented method produces an improved SNR and the low RMSE and PRD.
(3) The proposed method does not require any prior information and preserves the structural characteristics of ECG signals well.

The paper is organized as follow. Section 2 describes the theoretical basis and the presented time-frequency denoising framework. Section 3 demonstrates the effectiveness of the proposed method by comparing the denoising results obtained by wavelet transform based approach and NLM filtering. Section 4 presents the discussion. Section 5 concludes the work in this study.

## 2 Methodology

### 2.1 S-transform

The S-transform, first proposed by Stockwell et al. (27), can provide frequency-dependent resolution while maintaining a direct relationship with the Fourier spectrum, as well as the extraction of phases the compose the analyzed signal. In fact, the ST is a generalization of the short-time Fourier transform (STFT) by using a moving and scalable localizing Gaussian window. Moreover, it is similar to a continuous wavelet transform (CWT) in having progressive resolution and retains absolutely referenced phase information. Thus, it has found applications in a range of fields.

The ST of a signal x(t) is defined as follows:

(1) $$ST(t,f)=\int_{-\infty }^{\infty }x(\tau )g(\tau -t,f)e^{-i2\pi f\tau }d\tau$$

where *t* and *f* denote time and frequency, respectively. *τ* is a time parameter that controls the location of window function *g* in time, g(t,f) is Gaussian window function, which is represented as:

(2) $$g(t,f)=\frac{\left|f\right|}{\sqrt{2\pi }}e^{-\frac{t^{2}f^{2}}{2}}$$

The signal x(t) can be reconstructed from ST(t,f).


(3) $$x(\tau )=\int_{-\infty }^{\infty }\int_{-\infty }^{\infty }ST(t,f)e^{i2\pi f\tau }dtdf$$

### 2.2 Bi-dimensional empirical mode decomposition

**Figure 1:**
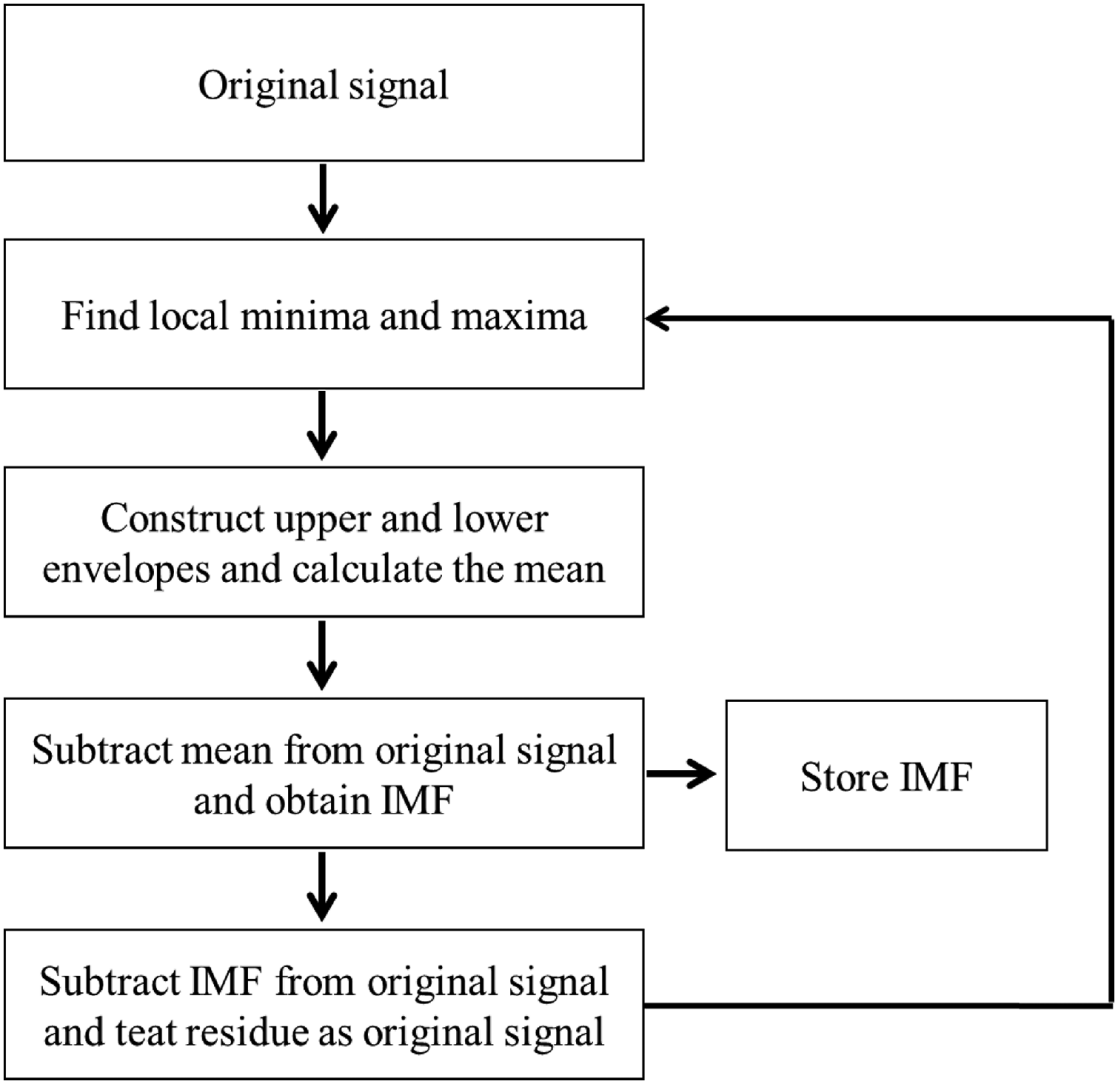
Flowchart of BEMD algorithm.

Bi-dimensional empirical mode decomposition (BEMD) (28) is an extended EMD in two dimensions (2D), where the iteration and sifting operations are the same with EMD. It can extract the different frequency components of image, and adaptively decompose a 2D signal into a set of bi-dimensional IMFs (BIMFs) with a residue. The core idea of the BEMD is to find the intrinsic multi-scale oscillations in the input signal, the details are described as follows (Figure 1):

(1) Search for all local minima and maxima of the 2D signal ST(t,f) based on cubic spline algorithm.
(2) Utilize the obtained the extrema to construct the maxima envelope $E_{\max}(t,f)$ and minima envelope $E_{\min}(t,f)$.
(3) Calculate the average envelope $avgE_{1}(t,f)$
  (4) $$avgE_{1}(t,f)=\frac{E_{\max}(t,f)+E_{\min}(t,f)}{2}$$
(4) Subtract the average envelope from the original signal ST(t,f)
  (5) $$e_{1}(t,f)=ST(t,f)-avgE_{1}(t,f)$$

Step (4) is repeated *n* times until the BIMF decomposition condition is met, thus, we have

(6) $$e_{1,n}(t,f)=e_{1,n-1}(t,f)-avgE_{1}(t,f)$$

(5) The first separated $BIMF_{1}$ and the residue are obtained
  (7) $$BIMF_{1}=e_{1,n}(t,f)$$
  (8) $$r_{1}(t,f)=ST(t,f)-BIMF_{1}$$
(6) The residue is treated as a new input of the sifting processing, and then the procedure is repeated *m* times until the stopping criterion is satisfied, so we obtain a set of BIMFs. Let $H_{ij}(t,f)$ represent the *j*th iteration in the *i*th sifting processing, thus
  (9) $$BIMF_{i}=ST(t,f)-H_{ij}(t,f)$$

### 2.3 Non-local means

Non-local means (NLM) filtering (29), originally proposed for image denoising (30), is a popular denoising algorithm, and it has been widely applied for noise removal in ECG signals. The key of the NIM algorithm is based on the self-similarity of the image. It estimates the value of the current pixel in the image by taking a weighted average of surrounding pixels with similar neighborhood structure. In fact, the time-frequency map reflects the evolution of signal energy over time and frequency, and the energy density between adjacent samples has a certain similarity. Therefore, the characteristics of time-frequency map are consistent with the one of the image required by NLM. In this study, the BIMFs are filtered by NLM to remove the noise at different scales.

Consider a noisy ECG signal

(10) y(n)=s(n)+υ(n)

where y(n) is the noisy signal, s(n) is the desired signal and υ(n) is the noise.

For a given sample s(m), the estimate $\hat{s}(m)$ is a weighted sum of values within a neighborhood N(m), namely

(11) $$\hat{s}(m)=\frac{1}{Z(m)}\sum_{n\in N(m)}\omega (m,n)y(n)$$

where $Z(m)=\sum_{n}\omega (m,n)$, and the weights are

(12) $$\begin{array}{rl} & \omega (m,n)=\exp\left(-\frac{\sum_{\delta \in \Delta }{\left(y(m+\delta )-y(n+\delta )\right)}^{2}}{2L_{\Delta }\lambda^{2}}\right)\\ & \;\;\;\;\;\;\;\;\;\;\;\;\;\;\;\;\;\;\;\;\;\;\;\;\;\;\;\;\;\;\;\;\;\;\;\;\;\;\;=\exp\left(-\frac{d^{2}(m,n)}{2L_{\Delta }\lambda^{2}}\right) \end{array}$$

where *λ* denotes a bandwidth parameter, Δ represents the local patch of samples surrounding *m*, which is composed of $L_{\Delta }$ samples, *d*^2^ is the sum of squared point-by point difference between samples of two patches that are centered on *m* and *n*, respectively.

It is worth noting that there are three key parameters that should be determined in the NLM algorithm, e.g., the bandwidth *λ*, the patch half-width *P* and the neighborhood half-width *Q*. The parameter *λ* controls the degree of smoothing, and the denoising performance mainly relies on the selection of *λ*. Specifically, a smaller *λ* will cause noise fluctuations while a larger *λ* will result in dissimilar patch to appear similarly. Vile and Kocher (31) suggested that the optimal bandwidth is 0.5*σ*, *σ* is the standard variance of noise. Tracey and Miller (32) suggested the parameter *Q* is equals to the high amplitude R-wave in QRS complex. The parameter *Q* is suggested to be large, but the computation cost will be raised.

### 2.4 Preprocessing

**Figure 2:**
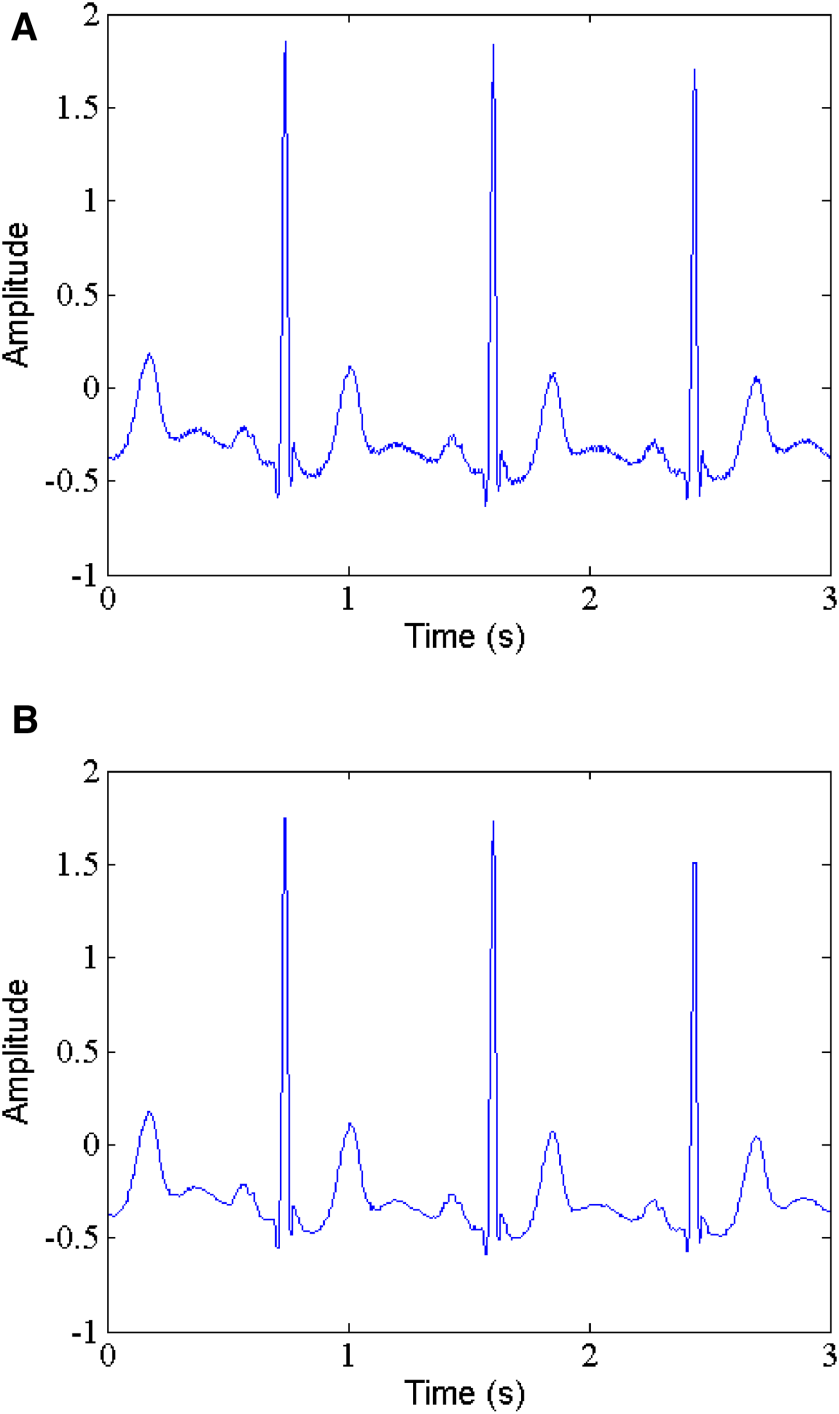
Raw noise removal, (**A**) noisy signal, (**B**) denoised signal.

The MIT-BIH arrhythmia database is a well-known publicly available data. It is worth noting that they contain the raw noise, not clean signals. Therefore, in order to evaluate the proposed method more fairly, we first employ median filtering to remove the raw noise. Figure 2 shows the original signal with raw noise (part of record 103) and the corresponding denoised signal by median filtering. As can be clearly seen, the raw noise has been effectively suppressed, thereby facilitating subsequent processing.

### 2.5 Proposed method

**Figure 3:**
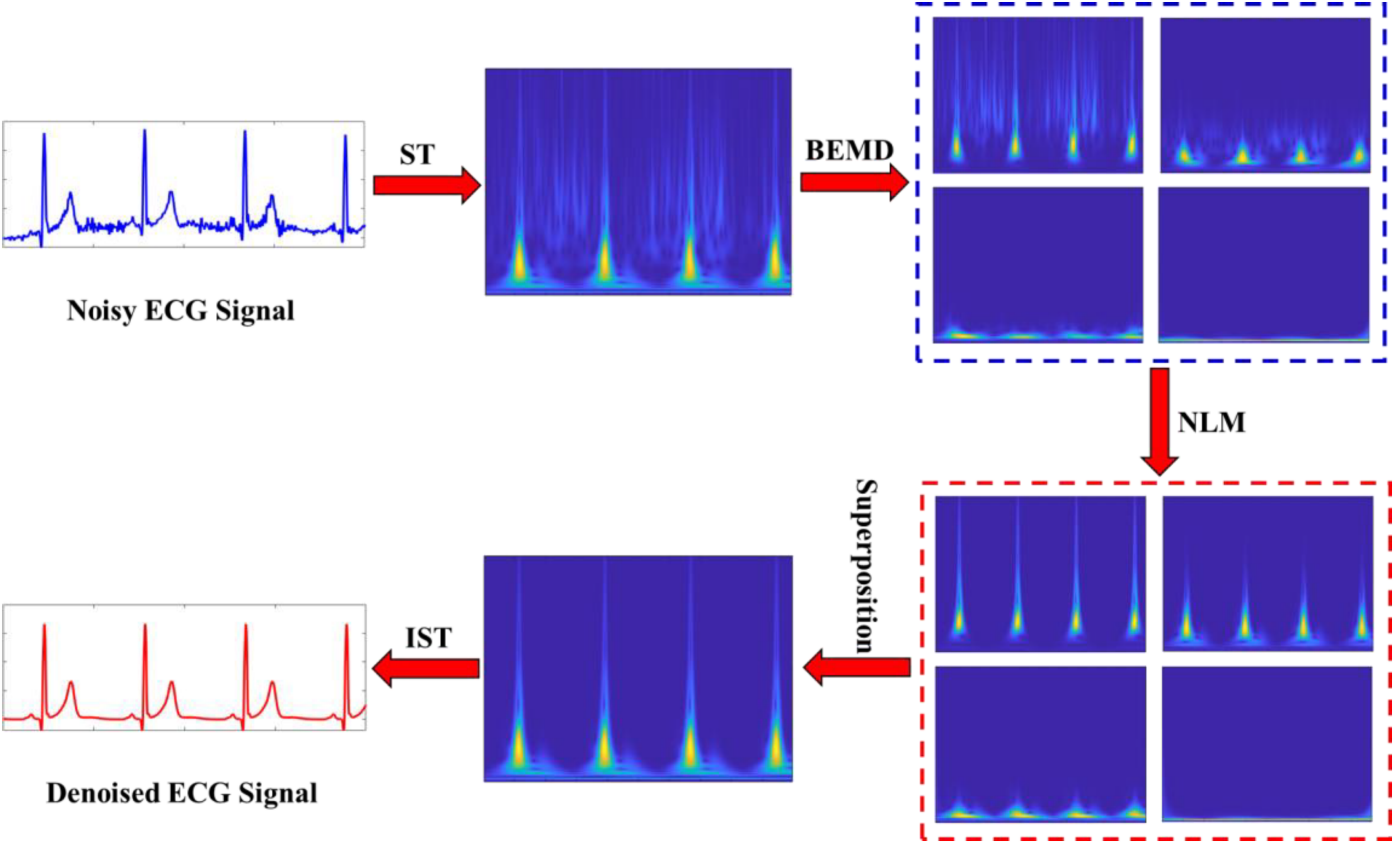
Flowchart of the proposed hybrid denoising scheme.

An efficient denoising method can reduce the impact of noise, so that the specialists accurately diagnose cardiac disease based on the extracted ECG characteristics. In this study, a hybrid denoising scheme in the time-frequency domain is proposed to denoise the ECG signals. Figure 3 shows the flowchart of the proposed method. The main steps are summarized as follows:

(1) Calculate the time-frequency representation (TFR) of an ECG signal using the ST method.
(2) Separate the magnitude spectrum and phase spectrum of the TFR.
(3) Decompose the magnitude spectrum of TFR using the BEMD algorithm into a set of BIMFs (including the residue component).
(4) Apply the NLM filer to each BIMF.
(5) Reconstruct the denoised magnitude spectrum by superimposing the processed BIMFs.
(6) Transform back the denoised magnitude spectrum into the time domain for recovering the denoised ECG signal by using the inverse ST (IST) method.

**Figure 4:**
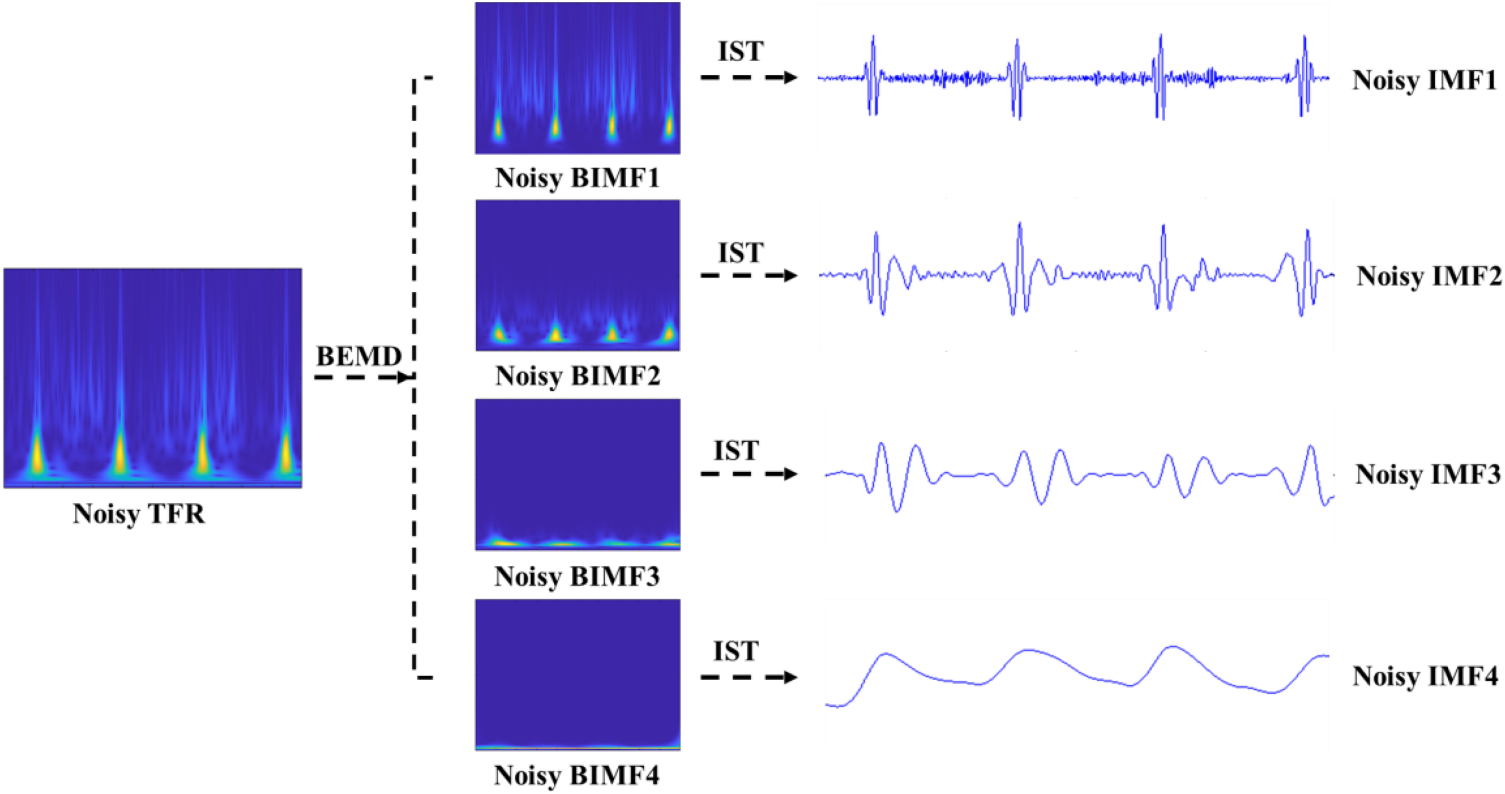
The noisy BIMFs decomposed by BEMD.

**Figure 5:**
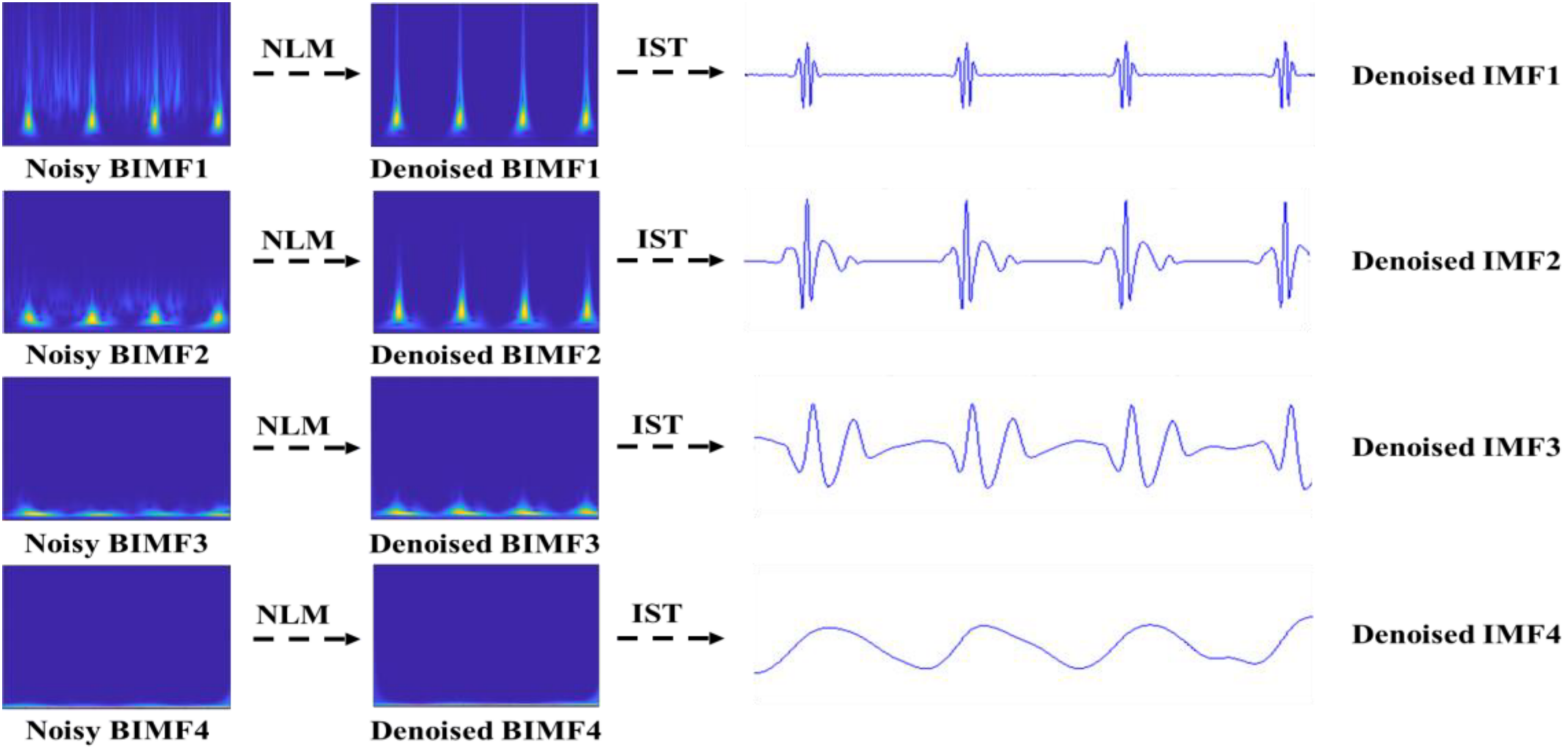
The denoised BIMFs by NLM.

The BEMD decomposition results are depicted in Figure 4. It can be clearly seen that a noisy time-frequency signal is decomposed into four BIMFs by BEMD algorithm, and the contents of frequency gradually decrease from BIMF1 to BIMF4. Then, we perform the IST on the noisy BIMFs to obtain the noisy time-domain IMFs. Similarly, the frequencies of time-domain IMFs are also gradually reduced from IMF1 to IMF4. Figure 5 shows the denoised BIMFs by the NLM filtering. The comparison between the noisy BIMFs and the denoised BIMFs indicates that most of noise has been successfully removed and the time-frequency energy is more concentrated. Also, we perform the IST on the denoised BIMFs and obtain the denoised time-domain IMFs. Compared with the noisy IMFs, the denoised IMFs are smoother, and ECG characteristics are significantly highlighted.

## 3 Results

The proposed method is evaluated on ECG signals collected from MIT-BIH arrhythmia database (33) and noise stress test database (34). The MIT-BIH arrhythmia database contains 48 records of 30 min each. They are digitized with a sampling frequency of 360 Hz with 11-bit resolution over a 10 mV range. We randomly choose the ECG signals from the database and extract 10 s length as the research object, then several types of noises are added to the ECG signals, which are PLI, Gaussian noise, BW, EM and MA, respectively. The performance of the proposed method is assessed against noises with a series of SNR levels (0, 5 and 10 dB), the denoised results are compared with the wavelet-based method and NLM filtering. In addition, the efficiency is quantitatively measured in terms of SNR, RMSE, PRD and SSIM, which are defined as follows.

(13) $$SNR=10\log_{10}\frac{\sum_{\;j=1}^{M}y_{j}^{2}(n)}{\sum_{\;j=1}^{M}{\left(y_{j}(n)-{\hat{y}}_{j}(n)\right)}^{2}}$$

(14) $$RMSE=\sqrt{\frac{1}{M}\sum_{\;j=1}^{M}{\left(y_{j}(n)-{\hat{y}}_{j}(n)\right)}^{2}}$$

(15) $$PRD=\sqrt{\frac{\sum_{\;j=1}^{M}{\left(y_{j}(n)-{\hat{y}}_{j}(n)\right)}^{2}}{\sum_{\;j=1}^{M}y_{j}^{2}(n)}}\times 100$$

(16) $$SSIM(x,y)=\frac{\left(2\mu_{x}\mu_{y}+C_{1})(2\sigma_{xy}+C_{2}\right)}{\left(\mu_{x}^{2}+\mu_{y}^{2}+C_{1})(\sigma_{x}^{2}+\sigma_{y}^{2}+C_{2}\right)}$$

where $y_{j}(n)$ is the original signal, ${\hat{y}}_{j}(n)$ is the denoised signal, *M* denotes the length of the ECG signal. *x* is the original image, *y* is the denoised image, $\mu_{x}$ and $\sigma_{x}$ are the mean and standard deviation of *x*, respectively, $\mu_{y}$ and $\sigma_{y}$ are the mean and standard deviation of *y*, respectively. $\sigma_{xy}$ is the covariance of *x* and *y*, $C_{1}=(K_{1}L{)}^{2}$, $C_{2}=(K_{2}L{)}^{2}$, *L* is the range of pixel values, and $K_{1}=0.01$, $K_{1}=0.03$.

It should be mentioned that the SNR measures the quality of the denoised ECG signal. The higher the output SNR, the better the denoising performance. The RMSE evaluates the variance between the real ECG signal and the denoised ECG signal. A lower RMSE means a smaller difference. The PRD indicates the distortion in the denoised ECG signal. A lower PRD represents a better recovery performance. The range of SSIM is between 0 and 1, and the larger the value, the better the quality of the image.

### 3.1 Power line interference

**Figure 6:**
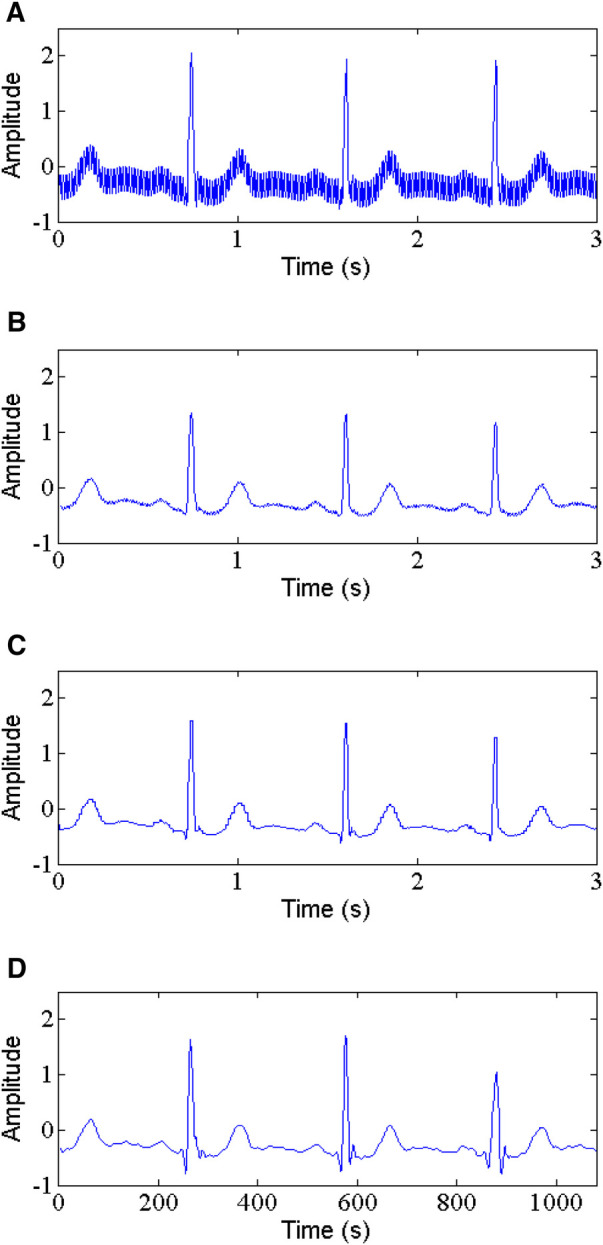
Power line interference removal, (**A**) noisy signal, (**B**) denoised signal by WT, (**C**) denoised signal by NLM, (**D**) denoised signal by the proposed method.

PLI is the most common noise with a frequency of 50 or 60 Hz and an amplitude of up to 50% peak-to peak ECG amplitude (35). In this study, the 60 Hz PLI is chosen as target. The comparison of the proposed method and some classic techniques such as WT-based method and NLM filtering for denoising PLI from ECG signals is shown in Table 1. As reported in Table 1, in removing PLI noise, the proposed method performs clearly well, with the higher output SNR, lower RMSE and PRD compared to WT-based method and NLM filtering. For example, for record 105, the proposed method produces an output SNR of 11.141 dB and a SSIM of 0.985, a RMSE of 0.0135 and a PRD of 30.96 at 5 dB input SNR (See Figure 6D). In contrast, the WT-based method (See Figure 6B) and NLM (See Figure 6C) obtain the output SNR of 5.059 dB and 9.560 dB, the RMSE of 0.0295 and 0.0283, the PRD of 44.15 and 38.48, and the SSIM of 0.708 and 0.876, respectively.

**Table 1:** A comparison of removing PLI at different input SNRs.

MIT/BIH tape No	Input SNR	PLI											
		Proposed method				WT				NLM			
		SNR	MSE	PRD	SSIM	SNR	MSE	PRD	SSIM	SNR	MSE	PRD	SSIM
105	0	8.043	0.0148	31.31	0.962	3.403	0.0957	79.43	0.668	6.994	0.0602	62.95	0.813
	5	11.141	0.0135	30.96	0.985	5.059	0.0295	44.15	0.708	9.560	0.0283	38.48	0.876
	10	14.171	0.0020	26.85	0.994	10.059	0.0193	34.83	0.802	12.628	0.0141	30.49	0.958
117	0	13.192	0.0059	7.25	0.969	3.045	0.1243	33.11	0.685	4.335	0.0919	28.46	0.703
	5	13.518	0.0055	6.98	0.984	5.004	0.0393	18.62	0.696	9.406	0.0143	11.23	0.844
	10	13.627	0.0051	6.70	0.991	10.004	0.0124	10.47	0.832	11.879	0.0064	7.535	0.854
212	0	12.276	0.0188	18.84	0.967	3.219	0.3342	79.43	0.606	4.145	0.3291	78.67	0.632
	5	12.555	0.0176	18.24	0.986	5.080	0.0986	43.15	0.747	6.913	0.0647	34.90	0.765
	10	13.637	0.0123	18.07	0.994	9.980	0.0319	24.54	0.875	11.522	0.0312	23.53	0.906
230	0	9.334	0.0153	26.70	0.962	3.353	0.1326	78.52	0.671	5.224	0.0997	68.00	0.713
	5	11.467	0.0148	26.39	0.984	5.064	0.0409	43.65	0.746	9.839	0.0337	40.22	0.885
	10	14.509	0.0142	26.16	0.994	10.064	0.0289	34.54	0.821	11.689	0.0214	31.43	0.934
232	0	9.488	0.0094	31.94	0.966	3.246	0.0844	95.49	0.664	4.405	0.0485	72.23	0.686
	5	11.626	0.0091	31.43	0.986	5.075	0.0260	53.08	0.710	10.559	0.0224	38.25	0.827
	10	13.669	0.0087	29.28	0.994	9.975	0.0184	34.19	0.868	12.731	0.0144	32.00	0.963

### 3.2 Gaussian noise

**Figure 7:**
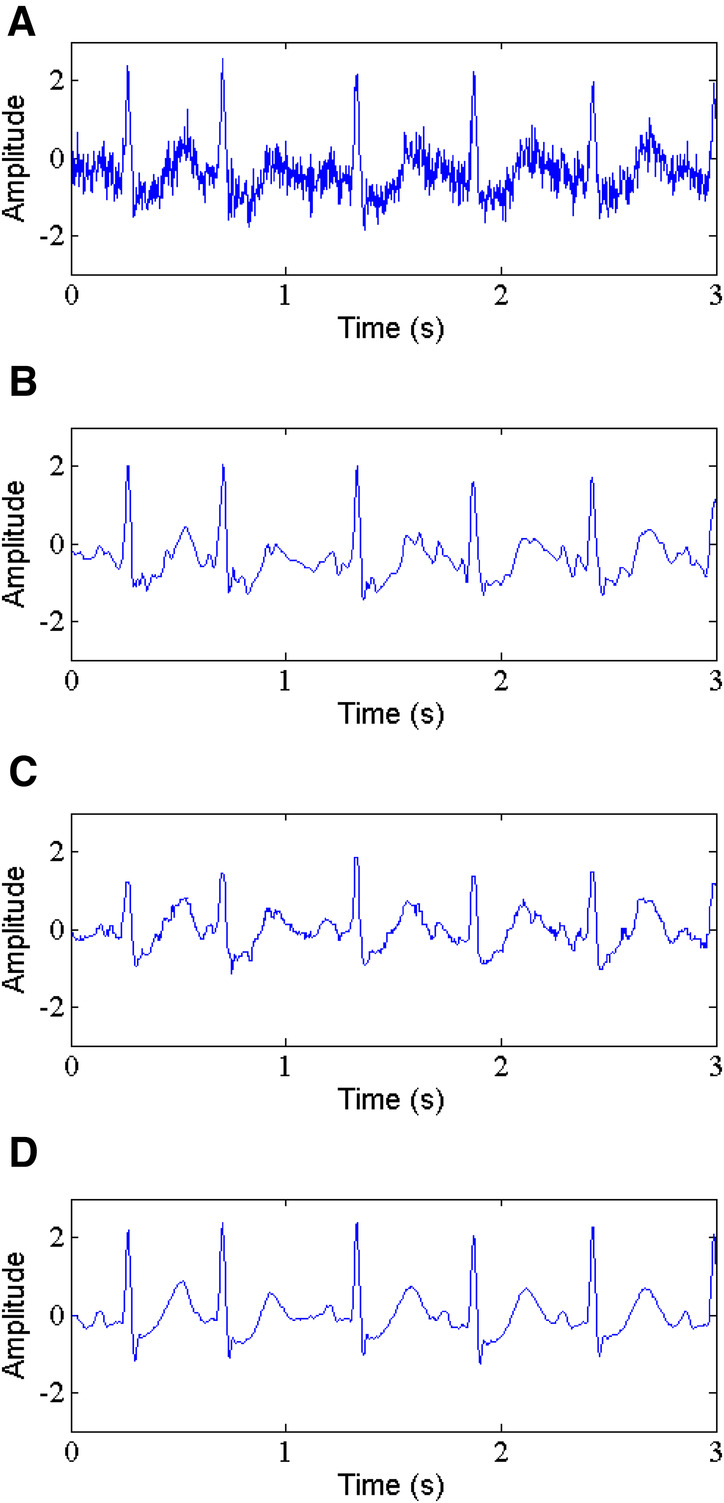
Gaussian noise removal, (**A**) noisy signal, (**B**) denoised signal by WT, (**C**) denoised signal by NLM, (**D**) denoised signal by the proposed method.

In Table 2, a comparison is shown for SNR, RMSE, PRD and SSIM of the proposed method and WT-based method and NLM filtering using five ECG records. The results demonstrate that the proposed method gives the higher values of output SNR and lower values of RMSE and PRD. For instance, for record 212 with input SNR of 5 dB and 10 dB (See Figure 7A), the proposed method (See Figure 7D) produces an output SNR of 13.296 dB and 16.099 dB, however, the WT-based method (See Figure 7B) and NLM filtering (See Figure 7C) give 10.107 dB, 11.783 dB and 9.074 dB, 10.112 dB, respectively. Similarly, the RMSE results also show better performance of the proposed method with the RMSE of 0.0297 and 0.0078, which are less than the values of the WT-based method and NLM filtering, which are 0.0309, 0.0210 and 0.0980, 0.0300, respectively. Moreover, the PRD values of the proposed method with 23.64 and 12.12 are the smallest among the comparison methods for the same record 212. In addition, the proposed method has the largest SSIM values, 0.967 and 0.976, respectively.

**Table 2:** A comparison of removing Gaussian noise at different input SNRs.

MIT/BIH tape No	Input SNR	White Gaussian Noise											
		Proposed method				WT				NLM			
		SNR	MSE	PRD	SSIM	SNR	MSE	PRD	SSIM	SNR	MSE	PRD	SSIM
105	0	8.642	0.0130	29.28	0.940	5.549	0.0263	41.73	0.692	4.232	0.0948	49.08	0.509
	5	12.843	0.0049	18.05	0.948	7.131	0.0183	31.78	0.856	5.032	0.0297	34.29	0.615
	10	13.715	0.0040	16.32	0.952	9.834	0.0156	32.08	0.943	10.047	0.0093	24.86	0.945
117	0	7.864	0.0324	16.89	0.831	7.054	0.1013	23.72	0.819	6.053	0.1229	32.92	0.783
	5	11.848	0.0081	8.48	0.890	10.043	0.0297	12.29	0.863	7.081	0.0385	18.45	0.864
	10	12.857	0.0044	7.55	0.898	10.54	0.0169	9.82	0.879	11.013	0.0123	9.46	0.891
212	0	8.409	0.0893	63.80	0.884	7.390	0.1576	71.07	0.814	6.001	0.3170	77.44	0.798
	5	13.296	0.0297	23.64	0.967	10.107	0.0309	34.19	0.906	9.074	0.0980	43.17	0.883
	10	16.099	0.0078	12.12	0.976	11.783	0.0210	19.94	0.942	10.112	0.0300	24.17	0.911
230	0	8.060	0.0226	38.96	0.933	6.186	0.0715	58.36	0.835	5.892	0.1286	67.37	0.761
	5	12.751	0.0070	18.03	0.945	8.167	0.0200	30.54	0.937	7.967	0.0419	44.14	0.909
	10	15.027	0.0041	13.87	0.957	10.044	0.0123	27.60	0.941	9.957	0.0132	28.85	0.914
232	0	8.755	0.0112	34.77	0.813	7.281	0.0197	46.20	0.796	6.815	0.0817	53.96	0.717
	5	11.596	0.0058	25.07	0.842	8.415	0.0120	36.14	0.807	8.043	0.0262	43.28	0.776
	10	12.533	0.0047	22.50	0.891	9.289	0.0090	32.68	0.863	10.003	0.0083	30.10	0.878

### 3.3 Baseline wander

**Figure 8:**
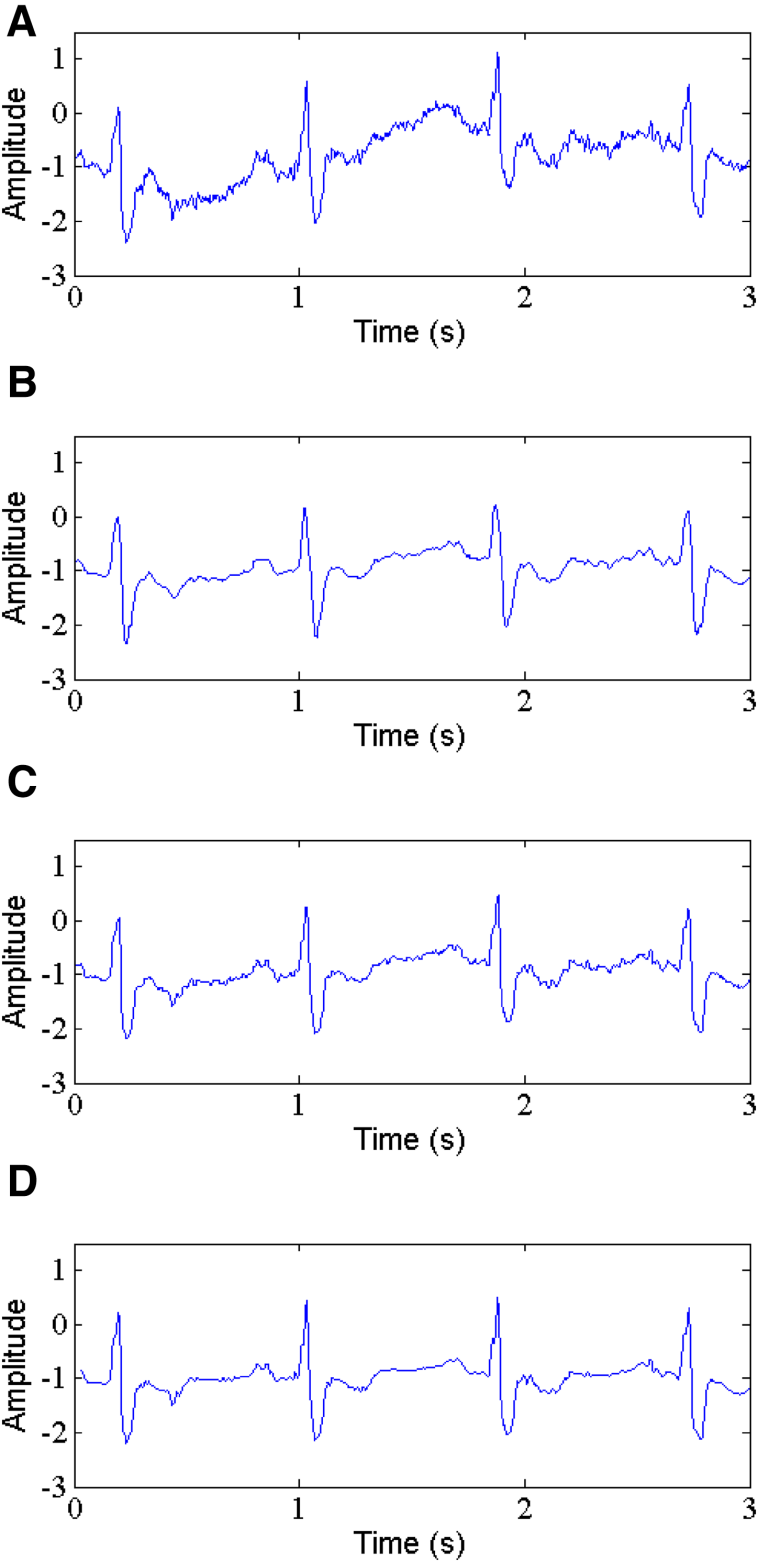
Baseline wander removal, (**A**) noisy signal, (**B**) denoised signal by WT, (**C**) denoised signal by NLM, (**D**) denoised signal by the proposed method.

Baseline wander is a low-frequency noise within 0.15–0.3 Hz with an amplitude of 15% of peak-peak ECG amplitude (15). It is very necessary to remove this noise from ECG signals, which makes interpreting ECG signal more difficult. The proposed method is applied to remove the BW, and we make a comparison with the other existing techniques, including the WT-based method and NLM filtering. Table 3 presents the values of parameters SNR, RMSE and PRD. The results show that the proposed method (See Figure 8D) provides better output results at the different input SNRs. For record 117, the output SNR and SSIM by our method is 14.001 dB and 0.972 when the input SNR is 10 dB, which is better than the WT-based method (See Figure 8B) and NLM filtering (See Figure 8C) by 8.454 dB, 0.752 and 8.055 dB, 0.727, respectively. For the same input SNR, the RMSE and PRD values for the selected ECG record 117 obtained by the proposed method are 0.0062, 10.47, respectively, which are lower than those of the WT-based method and NLM filtering with 0.0115, 12.51 and 0.0161, 13.43, respectively.

**Table 3:** A comparison of removing BW at different input SNRs.

MIT/BIH tape No	Input SNR	Baseline wander											
		Proposed method				WT				NLM			
		SNR	MSE	PRD	SSIM	SNR	MSE	PRD	SSIM	SNR	MSE	PRD	SSIM
105	0	4.7990	0.0062	64.27	0.540	0.9495	0.0622	85.20	0.018	1.411	0.0474	79.10	0.151
	5	8.0590	0.0147	34.15	0.737	3.336	0.0292	53.84	0.508	4.311	0.0246	43.97	0.509
	10	13.059	0.0046	24.83	0.924	6.011	0.0190	39.57	0.681	7.161	0.0096	34.72	0.723
117	0	1.3040	0.0421	30.11	0.262	0.4774	0.0695	43.81	0.014	0.551	0.0616	42.98	0.041
	5	9.0030	0.0196	14.62	0.815	4.452	0.0249	18.84	0.604	4.055	0.0284	19.54	0.603
	10	14.001	0.0062	10.47	0.972	8.454	0.0115	12.51	0.752	8.055	0.0161	13.43	0.727
212	0	4.8930	0.1595	57.62	0.510	0.7487	0.1768	75.70	0.003	0.6159	0.1882	77.22	0.004
	5	8.9800	0.050	33.65	0.751	4.295	0.0636	43.23	0.503	4.016	0.0790	47.42	0.483
	10	13.980	0.0159	24.54	0.927	9.107	0.0331	30.45	0.803	8.016	0.0458	34.42	0.704
230	0	4.6480	0.0647	67.62	0.501	0.660	0.0794	81.89	0.015	0.8178	0.0742	77.24	0.012
	5	8.0640	0.0204	33.79	0.751	3.736	0.0351	50.86	0.415	4.117	0.0303	43.43	0.510
	10	14.064	0.0064	24.54	0.967	6.778	0.0211	35.83	0.715	7.117	0.0164	34.42	0.780
232	0	4.241	0.0422	75.49	0.541	0.265	0.0712	100.46	0.011	0.479	0.0518	94.97	0.002
	5	8.9750	0.0133	43.70	0.812	3.712	0.0223	62.11	0.441	4.028	0.0202	53.40	0.463
	10	13.975	0.0042	20.19	0.925	6.820	0.0132	43.42	0.641	8.028	0.0130	30.03	0.783

### 3.4 Muscle artifact

**Figure 9:**
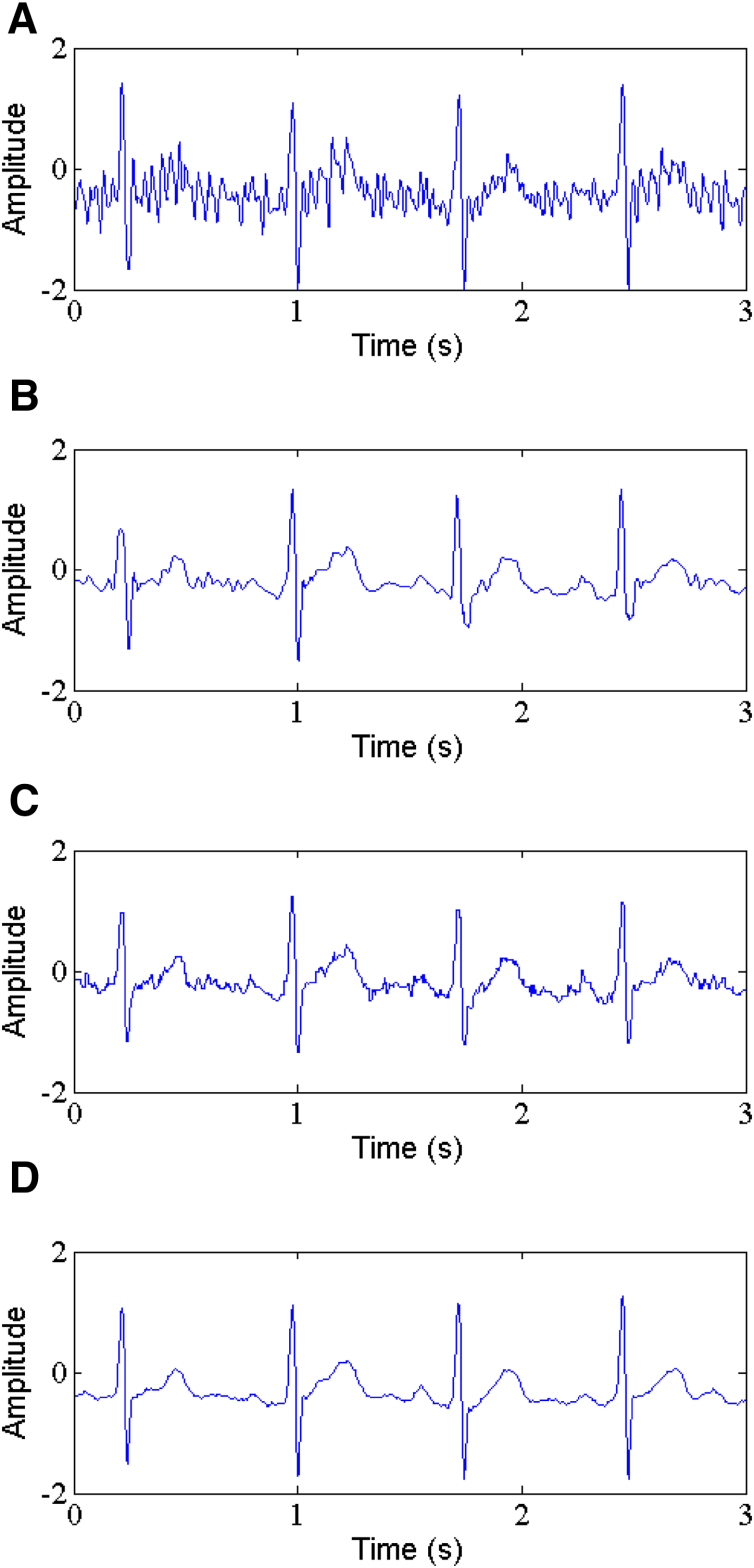
Muscle artifact removal, (**A**) noisy signal, (**B**) denoised signal by WT, (**C**) denoised signal by NLM, (**D**) denoised signal by the proposed method.

Muscle artifact with the low amplitude can mimic the baseline in atrial fibrillation that makes diagnosis difficult. Therefore, MA removal from ECG signals is a key task in the field of signal processing. The comparison of evaluation parameters such as SNR, RMSE, PRD and SSIM are listed in Table 4. It can be observed that the proposed method (See Figure 9D) brings a large improvement in the SNR, RMSE and PRD values compared with the WT-based method and NLM filtering. For example, the record 230 with an input SNR of 5 dB, the proposed method results (See Figure 9D) in an output SNR of 9.268 dB and a SSIM of 0.787, while the WT-based method (See Figure 9B) and NLM filtering (See Figure 9C) provide the output SNR and SSIM of 5.414 dB, 5.029 dB, and 0.370, 0.381, respectively. Similarly, Table 4 also indicates that the RMSE and PRD values obtained by the proposed method are 0.0045, 33.90. These values are lesser than the results from the WT-based method and NLM filtering, that is, 0.0175, 41.92 and 0.0208, 43.83, respectively.

**Table 4:** A comparison of removing MA at different input SNRs.

MIT/BIH tape No	Input SNR	Muscle artifact											
		Proposed method				WT				NLM			
		SNR	MSE	PRD	SSIM	SNR	MSE	PRD	SSIM	SNR	MSE	PRD	SSIM
105	0	6.793	0.0068	57.42	0.413	2.583	0.0209	68.88	0.241	3.076	0.0182	65.36	0.314
	5	9.111	0.0041	34.92	0.768	4.806	0.0163	45.46	0.358	4.988	0.0148	44.52	0.417
	10	11.76	0.0038	22.93	0.937	6.842	0.0150	35.96	0.605	8.972	0.0097	33.08	0.718
117	0	6.456	0.0099	25.01	0.817	3.293	0.0126	35.44	0.370	3.015	0.0318	36.18	0.354
	5	8.762	0.0063	15.23	0.923	6.545	0.0098	25.59	0.834	4.906	0.0189	28.83	0.804
	10	11.09	0.0029	10.38	0.983	8.060	0.0051	20.40	0.896	7.783	0.0060	23.74	0.875
212	0	5.311	0.0104	56.52	0.477	3.209	0.0371	64.05	0.429	2.433	0.1604	68.06	0.303
	5	9.138	0.0118	34.01	0.789	6.310	0.0337	43.45	0.689	4.990	0.0395	47.60	0.501
	10	12.736	0.0064	20.03	0.981	9.603	0.0190	32.63	0.898	9.911	0.0161	29.74	0.902
230	0	7.554	0.0089	48.34	0.435	1.780	0.0226	63.71	0.142	1.350	0.0657	77.88	0.119
	5	9.268	0.0045	33.90	0.787	5.414	0.0175	41.92	0.370	5.029	0.0208	43.83	0.381
	10	12.10	0.0036	21.79	0.957	8.749	0.0154	32.04	0.794	8.015	0.0162	34.68	0.784
232	0	6.512	0.0059	61.34	0.459	2.904	0.0136	76.48	0.276	2.079	0.0421	84.36	0.241
	5	8.593	0.0047	44.60	0.896	5.516	0.0106	50.45	0.367	4.991	0.0137	53.60	0.317
	10	11.730	0.0044	24.07	0.900	7.895	0.0095	38.37	0.808	8.022	0.0072	33.03	0.798

### 3.5 Electrode motion

**Figure 10:**
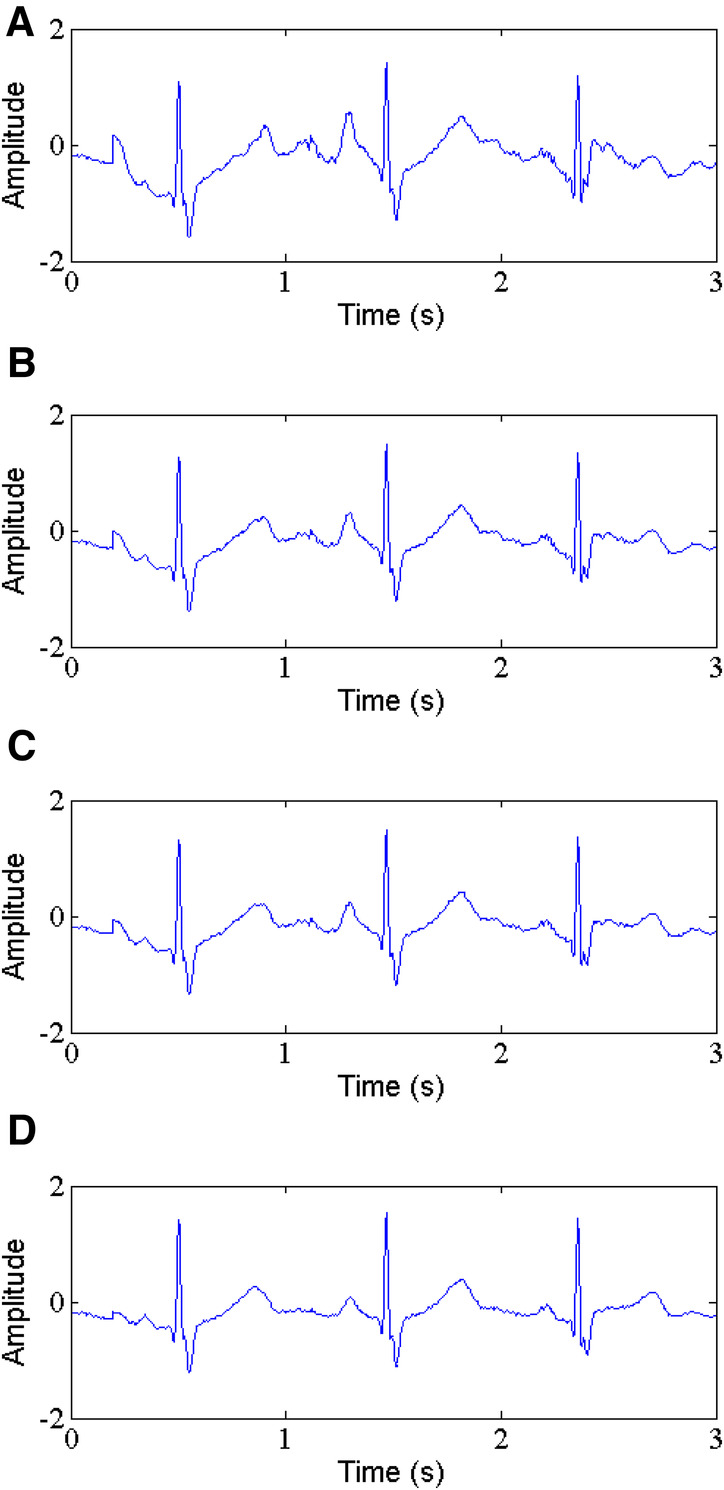
Electrode motion noise removal, (**A**) noisy signal, (**B**) denoised signal by WT, (**C**) denoised signal by NLM, (**D**) denoised signal by the proposed method.

Electrode motion noise is common in practice, and it can be mistaken for ectopic beat. However, it is very difficult to tackle using the traditional filtering techniques. Table 5 lists the values of parameters SNR, RMSE, PRD and SSIM of the denoised ECG signals obtained by the WT-based method, NLM filtering and the proposed method. The comparison indicates that the WT-based method and NLM filtering bring some improvements to some extent in terms of ECG signals enhancement. Nevertheless, the proposed method (See Figure 10D) does a better job, with higher SNR, SSIM and lower RMSE, PRD. It can be observed that the record 232 with an input SNR of 10 dB provides an output SNR and a SSIM of 12.520 dB, 0.950, respectively, while for the same record the WT-based method (See Figure 10B) and NLM filtering (See Figure 10C) provide the output SNR of 7.402 dB, 7.974 dB, and the SSIM of 0.861, 0.887, respectively. Similarly, the RMSE and PRD values given by the proposed method are 0.0047, 22.54, which are lower than those of the WT-based method and NLM filtering, that is, 0.0152, 32.61 and 0.0144, 31.20, respectively.

**Table 5:** A comparison of removing EM at different input SNRs.

MIT/BIH tape No	Input SNR	Electrode motion											
		Proposed method				WT				NLM			
		SNR	MSE	PRD	SSIM	SNR	MSE	PRD	SSIM	SNR	MSE	PRD	SSIM
105	0	4.373	0.0348	47.87	0.595	1.448	0.0855	75.08	0.104	1.345	0.0957	79.43	0.013
	5	11.408	0.0068	21.29	0.833	4.167	0.0363	48.93	0.587	5.059	0.0295	44.15	0.616
	10	13.698	0.0040	16.36	0.952	6.478	0.0213	37.50	0.862	8.058	0.0193	34.83	0.908
117	0	3.287	0.0586	22.73	0.444	1.361	0.0975	29.31	0.070	1.232	0.1243	33.11	0.003
	5	9.988	0.0125	10.51	0.896	5.575	0.0344	17.43	0.687	5.000	0.0393	18.62	0.634
	10	12.40	0.0071	7.96	0.966	9.313	0.0145	11.33	0.919	9.991	0.0124	10.48	0.924
212	0	4.059	0.1493	48.48	0.586	2.564	0.2545	69.31	0.315	2.016	0.3192	77.62	0.293
	5	8.281	0.0472	29.81	0.887	5.377	0.0921	41.70	0.677	4.980	0.1009	43.65	0.604
	10	13.480	0.0142	16.38	0.968	8.964	0.0403	27.59	0.906	10.078	0.0312	24.27	0.934
230	0	3.298	0.0618	53.55	0.573	2.696	0.1120	72.18	0.521	2.043	0.1311	78.07	0.482
	5	10.931	0.0106	22.24	0.937	4.609	0.0455	46.00	0.690	5.014	0.0414	43.90	0.0715
	10	14.552	0.0046	14.66	0.948	7.320	0.0243	33.66	0.891	10.013	0.0131	24.69	0.923
232	0	4.540	0.0296	56.49	0.530	2.694	0.0715	87.90	0.321	2.047	0.0844	95.50	0.262
	5	10.304	0.0078	29.09	0.844	4.648	0.0287	55.76	0.590	4.975	0.0267	53.70	0.603
	10	12.520	0.0047	22.54	0.950	7.402	0.0152	32.61	0.861	7.974	0.0144	31.20	0.887

## 4 Discussion

The non-stationary and nonlinear characteristics of ECG signals and various noise interferences make the analysis of ECG signals a very challenging task (36). The noise removal becomes an important research topic in ECG signals analysis. For ECG signals, it is necessary not only to analyze the frequency content of the signal, but also to characterize how the frequency changes with time.

The aim of this study is to address the problem of noise interference in the ECG signals by using an efficient hybrid denoising scheme in the time-frequency domain. We propose a new ECG signal denoising method by transforming a one-dimensional time-domain ECG signal into a two-dimensional time-frequency map using ST, BEMD and NLM approaches. The time-frequency map obtained by ST can better exhibit the characteristics of ECG signal and noise. BEMD can adaptively decompose the time-frequency signal into a series of BIMFs of different scales, thus, the characteristics of ECG signal and noise can be finely described at multiple scales. Subsequently, the noise can be removed at different scales by denoising the BIMFs using NLM algorithm based on structural self-similarity. Analysis of the results indicates that the proposed method can not only effectively suppress the noise in the ECG signals, but also better preserve the characteristics of ECG signals.

In the paper, two classic ECG denoising techniques including the WT-based method and NLM filtering are employed to compare the denoising performance. In our experiments, the proposed denoising scheme performs clearly better, with the higher output SNR and SSIM, lower RMSE and PRD for all the noises at different levels of input SNR. In fact, there are two important problems for the WT-based method (37). First, the filtered result depends on the selection of the mother wavelet, and it is difficult to find a suitable mother wavelet that is able to provide good filtered result in practice. Second, the WT may cause oscillation in the reconstructed ECG signal and the reduced amplitude of ECG waveform. The performance of NLM filtering relies on the selection of a parameter's bandwidth (38), which mainly depends on the noise standard deviation that might not be properly determined for ECG signals corrupted by a number of noises in the time domain.

The multi-scale time-frequency denoising method proposed in the paper exhibits outstanding performance. Our results show that the waveform variation trend of the signal after multi-scale time-frequency denoising fit well to the original signal with a smoother waveform. It is noteworthy that there are not too many parameters involved in the proposed algorithm, which makes it easier to embed into wearable ECG signal acquisition and analysis system as an effective complementary tool to traditional ECG denoising approaches in practice. In addition, there are several potential directions for future work. First, many image denoising algorithms and deep learning framework can help ECG signals analysis and processing in the multi-scale time-frequency domain. Second, time-frequency analysis is suitable for non-stationary and non-linear signals, thus, feature extraction and cardiovascular disease classification for ECG signals can be considered in the multi-scale time-frequency domain. In a word, the multi-scale time-frequency analysis of ECG signals has broad application prospects, and future work will focus on the combined use of multiple strategies to improve the quality of ECG signals and enhance the diagnosis accuracy of cardiovascular diseases.

## 5 Conclusion

In this paper, an efficient approach is proposed based on a hybrid scheme to remove various noises in ECG signals. The performance of the proposed method is investigated by comparing it with the existing techniques such as the WT-based method and NLM filtering. Experimental results on a wide variety of ECG signals demonstrate that the proposed method achieves the higher output SNR and SSIM and lower RMSE and PRD than the comparative methods for PLI, Gaussian noise, BW, MA and EM noise. The proposed method not only significantly suppresses the noise presented in ECG signals, but also preserves the characteristics of ECG signals better, thus, facilitating ECG signals analysis and processing for cardiovascular diseases detection.

## Data Availability

Publicly available datasets were analyzed in this study. This data can be found here: https://www.physionet.org/content/mitdb/1.0.0/.
